# Orthodontics and Obstructive Sleep Apnoea: Evaluating the Evidence on Airway Changes

**DOI:** 10.7759/cureus.90594

**Published:** 2025-08-20

**Authors:** Feras Y Dahhas, Ahmad A Baqer, Mona E Mitwalli, Suha M Alsharyoufi, Meshal H Alqarni, Raniya A Anze, Sahar L Alshammari, Hanan A Asiri, Abdulrahman S Khurayniq, Ali M Albrahim, Mohammed A Alessa

**Affiliations:** 1 Orthodontics, Al-Noor Specialist Hospital, Makkah, SAU; 2 Orthodontics, Jaber Al-Ahmad Al-Sabah Hospital, Kuwait, KWT; 3 Dentistry, Batterjee Medical College, Jeddah, SAU; 4 Dentistry, King Abdulaziz University, Jeddah, SAU; 5 Dentistry, Vision College, Riyadh, SAU; 6 Dentistry, University of Hail, Hail, SAU; 7 Dentistry, Armed Forces Hospital Southern Region, Khamis Mushait, SAU; 8 Dentistry, Taibah University, Medina, SAU; 9 Dentistry, Imam Abdulrahman Bin Faisal University, Dammam, SAU

**Keywords:** airway obstruction, craniofacial morphology, mandibular advancement devices, obstructive sleep apnoea (osa), orthodontic treatment

## Abstract

Obstructive sleep apnoea (OSA) is the recurring folding of the upper airway during sleep, resulting in complete or partial airway obstruction leading to apnoea, hypopnea, or both. Left undiagnosed and untreated, it can lead to several complications; for instance, neurocognitive dysfunction, such as dizziness and impaired quality of life, due to reduced sleep and increased fatigue, as well as cardiovascular diseases such as hypertension, stroke, myocardial infarction, and heart failure. Obstructive sleep apnoea is generally diagnosed in a sleep laboratory over the course of a night and can also be detected at home following alternative approaches. In addition to the conventional treatment modalities such as adenotonsillectomy and continuous positive airway pressure, orthodontists play a vital role in the management of OSA via a multidisciplinary approach. Specifically, orthodontists can influence craniofacial anatomy through both surgical and non-surgical approaches. This review aims to establish the relationship between orthodontic interventions, both surgical and non-surgical, and the management of OSA. In addition, it explores the pathophysiology and diagnosis of OSA, various treatment options, and the use of novel technology to assist in the management of OSA.

## Introduction and background

Obstructive sleep apnoea (OSA) is the recurring folding of the upper airway during sleep. This condition results in the complete or partial obstruction of the airway and leads to apnoea, hypopnea, or both. Apnoea is defined as the termination of airflow for 10 seconds or more, whereas hypopnea refers to a reduction of airflow by 30% for 10 seconds with a 4% decrease in oxygen saturation [1]. The anatomic aetiology involves small upper airways in conjunction with the pharyngeal dilator muscles that compensate for the anatomic defect while awake, but not asleep [2]. Thus, the anatomically compromised upper airway causes sleep-induced loss of compensatory tonic input to the upper-airway dilator muscle motor neurones, leading to collapse of the pharyngeal airway. As mentioned earlier, apnoea and hypopnea are restricted to sleep and may be obstructive (complete closure of the extrathoracic upper airway), central (marked reduction or cessation of brainstem respiratory motor output), or mixed [3].

If left undiagnosed and untreated, several complications can occur. For example, patients may encounter neurocognitive dysfunction (dizziness and impaired quality of life) due to the lack of sleep and increased fatigue. The intermittent hypoxia may also cause cardiovascular diseases, such as hypertension, stroke, myocardial infarction, and heart failure [2]. Thus, an accurate and timely diagnosis is important for effective management of this condition.

OSA is generally diagnosed in a sleep laboratory over the course of a night, but it can also be detected at home following alternative approaches. However, the close association of pharyngeal muscles with the oral musculoskeletal structures [4] warrants the involvement of orthodontists and maxillofacial surgeons to assess the patient as part of a multidisciplinary approach to management. Accordingly, orthodontists have found that soft tissue examination, including facial contours, neuromuscular function, tongue, tonsils, adenoids, and nasal polyps, forms an integral part of accurate diagnosis and treatment planning [5]. Orthodontists also consider skeletal discrepancies such as mandibular retrusion, maxillary constriction, and vertical facial excess, as these can directly contribute to airway narrowing and OSA severity. Identifying these craniofacial patterns early allows for interceptive treatment, which may include maxillary expansion, mandibular advancement devices (MADs), or orthognathic surgery in severe cases [1-5]. Several treatment options are available to treat this condition, ranging from non-surgical therapy to surgical procedures. Continuous positive airway pressure (CPAP) remains the most sought-after treatment approach. That said, it depends on the individual patient's needs and the severity of the condition, as some patients may have to use oral appliances (e.g., to advance the mandible) or undergo surgical procedures [2]. Due to the variety of different treatment options and to facilitate prompt treatment, a multidisciplinary approach is necessary [1].

As airway anatomy is closely related to craniofacial structures, features such as a narrow maxilla and a retruded mandible can contribute to airway obstruction [2]. Therefore, this review aims to establish a relationship between orthodontic treatment and its impact on OSA. It discusses in detail the different orthodontic treatment modalities used to improve OSA, as well as their effectiveness in adult and paediatric patients. Due to growing clinical interest and emerging research suggesting that orthodontic interventions can improve respiratory function as well as airway dimensions, this review examines the impact of orthodontic treatments on airways and OSA. Moreover, as OSA is associated with cardiovascular diseases, metabolic disorders, neurocognitive impairment, and low quality of life, it is a serious health concern that necessitates prompt management. This review will provide practitioners with an overview of OSA as a significant health concern that affects both children and adults.

## Review

Methodology

This narrative review presents a comprehensive synthesis of both established and emerging research related to OSA and its treatment modalities in paediatric and adult populations. An extensive electronic literature search was conducted across several databases, including PubMed, ScienceDirect, Scopus, Google Scholar, and Cureus, covering publications from 2000 to 2024. The search strategy employed keywords such as “Obstructive Sleep Apnoea”, “OSA”, “Upper Airway”, “Orthodontics”, “Orthognathic Surgery”, and “Paediatric Sleep Disorders” combined using Boolean operators to improve specificity and relevance.

Inclusion Criteria

Studies were included if they were peer-reviewed, published in English, and focused on orthodontic or orthognathic surgical interventions and their impact on airway dimensions or OSA outcomes. Both narrative and systematic reviews, clinical trials, and cohort studies were considered, provided they had clearly stated methodologies and reported outcomes. Articles addressing diagnostic tools, surgical techniques, or orthodontic modalities for the management of OSA in either paediatric or adult populations were also included.

Exclusion Criteria

Studies were excluded if they were published in languages other than English, consisted only of abstracts without full text, or lacked a clear methodological framework. Editorials, opinion pieces, case reports, and studies unrelated to orthodontic, surgical, or airway-focused management of OSA were also excluded.

Although this review does not follow a formal Preferred Reporting Items for Systematic Reviews and Meta-Analyses (PRISMA) flowchart due to its narrative nature, a structured and systematic selection process was implemented. After removing duplicates, titles and abstracts were screened for relevance, followed by full-text reviews to ensure that only high-quality, pertinent studies were included in the final analysis.

Discussion

Pathophysiology of OSA and Risk Factors

As mentioned earlier, OSA occurs due to the collapse of the upper airway. There are several anatomic risk factors for OSA; for instance, tongue size, tongue fat, and pterygomandibular fat have been linked with the Apnoea Hypopnea Index (AHI) [6,7]. Tongue fat leads to a larger tongue, with potentially reduced force, resulting in functional loss as an upper airway dilator. Extended tonsillar spaces have also been associated with OSA in both children and adults [8,9]. In addition, craniofacial anatomical variations such as a narrow maxilla, a shortened mandible, and a long face also influence the patency of the upper airway in both growing and non-growing individuals [10,11]. A narrow maxilla and high-arched palate in patients with OSA contribute to elevated nasal resistance and posterior displacement of the tongue [11]. In children, the pathophysiology of OSA is based on multiple contributing factors, such as adenotonsillar hypertrophy, obesity, neuromuscular disorders, and craniofacial abnormalities. Furthermore, research confirms that in children, the peak of OSA can be observed around 10 years of age, when the lymphatic system is at its most developed [12].

Symptoms of OSA

Paediatric patients: The estimated prevalence of OSA in children ranges between 1% and 4%, depending on the methods used to diagnose the condition [4]. The most common symptoms of OSA in children are persistent snoring often accompanied by breathing pauses, paradoxical or difficult night breathing, sleeping disorders with recurrent night awakenings [8,13], excessive night sweating, and sporadic enuresis [8,13]. Other symptoms that occur at night are nightmares, agitation, and attaining a posture that causes the neck to extend greatly. During the day, children may experience long hours of day sleep, headaches after waking up, irritability, and poor academic performance [13].

Adult patients: Similarly, adults also experience fragmented night sleep with loud, interrupting snoring, apnoea, and excessive daytime sleepiness, leading to poor quality of life and impaired mental health [14]. Patients with OSA are often obese (BMI > 28 kg/m^2^). They also tend to have a large neck circumference (> 40 cm), nasal problems (deviated nasal septum), an elongated and low-lying uvula, enlarged tonsils and adenoids, macroglossia, dental malocclusion, a narrow mandible, a narrow maxilla, retrognathia, and a high and narrow hard palate, etc. [15]. Table 1 summarises the key symptoms of OSA observed during sleep and while awake in both children and adults, offering a side-by-side comparison for easier clinical reference.

**Table 1:** Symptoms of Obstructive Sleep Apnoea During Sleep and While Awake in Children and Adults

Patient Group	Symptoms During Sleep	Symptoms While Awake
Children	interrupting snoring [12]. Breathing pauses [13]. Night sweats [13]. Enuresis (bedwetting) [13]. Nightmares [13]. Unusual sleeping postures [13]. (e.g., extended neck). Restless sleep [14].	Daytime sleepiness [15]. Headaches upon waking [15]. Irritability [15]. Difficulty concentrating [15]. Poor academic performance [15].
Adults	Gasping or choking during sleep [1,2]. Loud and disruptive snoring [14]. Apnoea episodes [14]. Frequent awakenings [14].	Excessive daytime fatigue [14]. Impaired mental health [14].

Diagnosis of OSA

A thorough patient evaluation, comprising history taking and clinical examination, is necessary to diagnose OSA in a sleep clinic. However, these can only predict OSA in 50% of the patients. To establish a definitive diagnosis, it is imperative to study the patient’s breathing patterns overnight [15]. One of the most important variables used to assess the severity of the OSA is the AHI. The AHI value represents the average number of apnoea and hypopnea events per hour of sleep. The classification of severity in adults is given as follows: normal (AHI < 5), mild (5 ≤ AHI < 15), moderate (15 ≤ AHI < 30), or severe (AHI ≥ 30) [11].

Different methods can be used to identify OSA in an individual. Some studies have discussed nocturnal sleep laboratory-based polysomnography (PSG) and in-home sleep studies using portable sleep monitor devices [9,12]. Additionally, previous studies have discussed the utility of patient-reported and parent-reported questionnaires in investigating the outcomes achieved by adenotonsillectomy, a surgical procedure used to treat OSA [10]. Although the patient’s history plays an essential role in the evaluation process, sleep laboratory-based PSG should be used as the gold standard for the diagnosis of OSA through the assessment of the AHI [12].

Chervin et al. compared the use of PSG with a questionnaire approach to predict improvement after adenotonsillectomy; their study concluded that the questionnaire method was not as reliable as the former approach for individual patients [10]. Despite its vast availability in sleep-disorder clinics in developed countries, this method is not typically well-received by patients [12]. Instead, portable and home sleep tests have been suggested as an alternative option to PSG for the screening of OSA, particularly in children. Furthermore, three-dimensional (3D) imaging techniques such as cone-beam computed tomography (CBCT) are potential diagnostic methods to analyse airway dimensions because of their ability to determine the boundaries between soft tissues and air spaces [4]. To monitor outcomes after introducing a course of treatment to the patient, changes in AHI, oxygen saturation levels, polysomnographic variables, cephalometric factors, nasal parameters, upper airway dimensions, and clinical symptoms should be keenly observed [16].

Management of OSA

There are various treatment modalities available to treat OSA depending on the aetiology of the airway obstruction and the severity of the condition.

Adenotonsillectomy: The most frequent cause of OSA remains adenotonsillar hypertrophy, and the suggested treatment strategy for this is adenotonsillectomy. Following that, a PSG check is scheduled to check if OSA is still present [13]. However, the success of surgical treatment is not guaranteed, and up to 68% of people show post-surgical relapse, especially when there is an underlying skeletal structure issue, increased soft tissue crowding of the oropharynx, or increased BMI [12]. Table 2 provides a simplified overview of the most commonly used treatment approaches for OSA, highlighting how each method works and when it is typically recommended depending on the severity of the condition and patient suitability.

**Table 2:** Management Options for Obstructive Sleep Apnoea OSA: Obstructive Sleep Apnoea; BMI: Body Mass Index

Treatment Approach	How It Works	When It’s Usually Used
Adenotonsillectomy (AT)	Removes enlarged tonsils and adenoids that block the airway [13].	First-line treatment for children with OSA caused by adenotonsillar hypertrophy [13-26].
CPAP (Continuous Positive Airway Pressure)	Keeps airways open by gently blowing air through a mask [14].	Used for moderate to severe OSA, especially when surgery isn’t an option or hasn’t worked [14].
Mandibular Advancement Devices (MADs)	Moves the lower jaw forward during sleep to keep the airway open [19].	Recommended for mild to moderate OSA, or for those who can’t tolerate CPAP [19]
Rapid Palatal Expansion (RPE)	Widening the upper jaw to open up nasal passages and make more room for the tongue [13,18,19].	Often used in children with narrow maxilla and breathing issues during sleep [13,18,19].
Orthognathic Surgery	Surgically repositions the jaws to enlarge the airway [18].	Considered for severe OSA or cases with significant jaw discrepancies [18].
Hypoglossal Nerve Stimulation	Stimulates the tongue muscle to prevent it from blocking the airway [18].	Used in adults with moderate to severe OSA who don’t respond to CPAP [18].
Leukotriene Antagonists & Nasal Steroids	Reduce airway inflammation and swelling [13].	Helpful for children with mild OSA or used alongside other treatments [13].
Bariatric Surgery	Helps with weight loss to reduce fat around the airway [13].	Considered in obese adolescents or adults with OSA and high BMI [13].

Nasal CPAP: CPAP is often a successful treatment option even among younger children [13]. It is most suitable for patients with moderate to severe OSA. With strong compliance, significant improvements have been observed through the reduction of nocturnal apneas and hypopneas. However, maintaining consistent adherence remains a challenge, and healthcare providers seek alternative approaches [1]. Therefore, it is not recommended to use CPAP as a first-line treatment; it should only be reserved for patients who have not responded to adenotonsillectomy or do not meet the surgical criteria [13].

Bariatric surgery:* *Obesity is a common clinical feature seen in patients with OSA [13]. Consequently, weight loss is often associated with improvements in both obesity and OSA. Additionally, losing weight reduces the risk of cardiometabolic abnormalities related to these conditions. However, achieving and maintaining weight loss can be challenging. Alternatively, bariatric surgery is used as a treatment for extremely obese patients and those with cardiovascular disturbances. Positive outcomes have been observed in managing sleep apnea, but a multidisciplinary approach is necessary for complete remission [11]. In 2012, guidelines were released for treating severe obesity with bariatric surgery in adolescent patients. Several studies have shown that gastric banding, gastric bypass, and sleeve gastrectomy effectively lower AHI scores and BMIs [13].

Leukotriene antagonists: Several studies have reported the effectiveness of leukotriene antagonists such as montelukast and topical nasal corticosteroids like fluticasone and budesonide in treating mild OSA [12,13]. Zaffanello et al. reviewed the safety and efficacy of intranasal corticosteroids and oral montelukast for OSA and adenoid hypertrophy in otherwise healthy children [12]. They concluded that combination therapy with montelukast and intranasal corticosteroids was more effective than using intranasal corticosteroids alone, such as mometasone, beclomethasone, budesonide, fluticasone, and flunisolide. However, they remain safer and effective options for managing mild to moderate OSA in children [12].

Orthodontic Treatments

There are a few orthodontic options available to help manage OSA. As pointed out earlier, craniofacial anatomical features such as a narrow maxilla and a retruded mandible have been found to negatively impact the dimensions of the upper airway. Thus, there is growing evidence that orthodontic management of the patient, whether paediatric or adult, has a positive impact on the already existing condition. For instance, rapid maxillary expansion produces transverse changes in narrow arch dimensions by widening it. Mandibular advancement leads to anterior displacement of the lower jaw, which increases the upper airway spaces and decreases the susceptibility of the pharyngeal airway to collapse. As shown by different studies, both of these treatment modalities work synergistically to lead to changes in airway dimensions [17].

Rapid Palatal Expansion (RPE)

RPE is a widely utilized intervention for paediatric patients with OSA, particularly those with a constricted upper jaw. This approach addresses the transverse deficiency of the maxillary arch, which, if uncorrected, can contribute to airway obstruction during sleep [13]. RPE involves the use of a fixed appliance equipped with an expansion screw, typically worn for three to four months. The appliance functions by reopening the mid-palatal suture, thereby widening the hard palate through the displacement of circummaxillary sutures across all three spatial planes [13,18]. This expansion reduces nasal airflow resistance and facilitates tongue repositioning, ultimately lowering the risk of airway obstruction associated with sleep apnoea [19].

Caruso et al. conducted a clinical study involving 14 paediatric patients with Class III malocclusion and maxillary constriction who also exhibited OSA symptoms [20]. The patients received RPE therapy in conjunction with Delaire’s face mask. Cephalometric analysis performed before and after treatment demonstrated a significant enhancement in linear measurements of the upper airway, including oropharyngeal and nasopharyngeal dimensions, indicating improved airway patency and alleviation of OSA symptoms.

Similarly, Pirelli et al. evaluated skeletal changes following RPE in children with OSA using low-dose computed tomography (CT) [21]. They observed notable improvements in first molar angulation, maxillary base width, nasal cavity width, and reopening of the mid-palatal suture after treatment. In a retrospective study by Yoon et al., the effectiveness of RPE was assessed in 60 children with OSA (mean age: eight years) [22]. Participants were divided into a treatment group and a control group. Results showed a marked reduction in palatine tonsil and adenoid volumes in the RPE group compared to controls.

Further evidence by Kim et al. confirmed substantial improvements in respiratory function among 26 children treated with RPE [23]. All cases exhibited enlargement of the nasomaxillary complex and notable reductions in snoring and AHI scores. In a later study, Pirelli et al. investigated nasal airflow changes post RPE in 19 children [24]. CBCT scans and clinical evaluations revealed significant increases in the volume of both the nasal cavity and nasopharynx. This anatomical expansion translated into reduced snoring and enhanced nasal breathing efficiency.

As demonstrated by a few studies that were followed up for 10-12 years, the literature supports the long-term effectiveness of RPE in patients with narrow maxilla and its success against OSA [13]. However, a study by Li et al. discusses an alternative approach for patients previously treated with RPE [25]. Skeletally fixed transpalatal distraction (TPD) was used by them to produce nasomaxillary expansion in patients with residual OSA. The adjunctive treatment further reduced the apnoea episodes, opened the mid-palatal suture, and widened the nasal passages with improved airflow in the nasal and pharyngeal airways as confirmed by polysomnographic results.

While RME has shown beneficial effects in managing OSA, the American Association of Orthodontics maintains that current evidence does not yet support its routine use as a preventive measure for paediatric OSA. They recommend that RME be reserved primarily for cases with transverse maxillary deficiency [18]. Additionally, a randomized clinical trial conducted by Magalhães et al. evaluated the impact of adenotonsillectomy versus RME on the AHI score and upper airway volume in 39 children with narrow maxilla [26]. Their findings indicated that children treated with adenotonsillectomy experienced a more substantial reduction in AHI and greater airway volume increase. These results support adenotonsillectomy as the first-line treatment for paediatric OSA, with RME serving as a complementary approach.

Mandibular Advancement (Non-Invasive)

Mandibular advancement is an orthopaedic strategy designed to reposition the mandible forward, particularly in patients with dental or skeletal retrognathia [14,19]. By encouraging the mandible to grow in a more anterior and downward direction, this approach helps enlarge the airway both during sleep and wakefulness. It is especially beneficial for individuals with mild to moderate OSA who are not candidates for surgical jaw repositioning or who have not responded to initial treatments like adenotonsillectomy or CPAP therapy [19].

MADs are intraoral appliances worn during sleep that bring the lower jaw forward to prevent airway obstruction [15]. These devices must be removed upon waking. Their action resembles that of functional appliances used in treating Class II malocclusion, as they promote forward movement of the tongue, soft palate, and hyoid bone, increase the lateral space of the velopharynx, and activate upper airway dilator muscles. MADs are typically recommended for patients with mild to moderate OSA [27].

Several studies have examined the role of functional appliances in managing OSA. Ganesh et al. noted improvements in oropharyngeal and hypopharyngeal space following the use of fixed functional appliances [27]. For patients intolerant of CPAP, MADs can also be considered for severe cases. These devices help decrease snoring, enhance sleep quality, and improve overall well-being. Their success is typically measured by either a 50% or more reduction in the AHI score or a drop in OSA severity classification (e.g., from moderate to mild) [28]. Research by Sutherland et al. [28] and Marklund et al. [29] revealed that although MADs might not eliminate OSA entirely, they can substantially lower AHI scores and boost oxygen saturation during sleep [28,29].

Before fabricating a MAD, a bite registration is taken with the mandible advanced to about 50-75% of its maximum protrusive range [11]. MADs may be prefabricated or custom-made and come in mono-block (fixed) or bi-block (adjustable) formats. The process of titrating gradual mandibular advancement over time is crucial to optimizing therapeutic outcomes. Custom, titratable appliances tend to offer greater comfort and better outcomes, particularly in moderate to severe OSA. Treatment progress is monitored through follow-up PSG, evaluating both snoring frequency and overall sleep quality.

Given that MADs are tooth-supported, rigid acrylic designs are preferred for their stability, although they may be slightly less comfortable than acetate alternatives. Their long-term use is associated with fewer dental side effects when appropriately monitored [18]. A 10-year study by Venema et al. confirmed the effectiveness of both MADs and CPAP in reducing OSA symptoms over time [30].

Potential side effects of MADs include dry mouth or excessive salivation, temporomandibular joint (TMJ) discomfort, and changes in bite [31]. Patients with pre-existing TMJ issues should be evaluated by a specialist before commencing MAD therapy. Over time, some users may experience changes in dental alignment, such as forward movement of lower incisors and retroclination of upper incisors, leading to a decreased overjet and overbite [32]. According to Knappe et al., temporomandibular disorders (TMD) symptoms reported included joint sounds such as clicking and crepitus [33]. They concluded that MADs might induce changes in the TMJs, disrupt occlusal harmony, and alter oro-facial function. Doff et al. reported an average 1.5 mm reduction in overjet over a two-year use period [32]. Although these changes are generally minor, regular dental follow-up is advised, and treatment modifications may be necessary [33,32].

Recent literature suggests that combining MADs with RME in growing patients may yield additional benefits, though whether this dual approach offers better long-term outcomes than RME alone remains uncertain. MADs are effective during the active treatment phase but typically do not result in lasting changes to mandibular length or position in growing individuals [34].

MADs, such as the Herbst appliance, have demonstrated improvements in both oropharyngeal and hypopharyngeal airway dimensions [35].Similarly, twin block appliances have been successfully utilised in paediatric patients, with some studies reporting up to a 37% reduction in AHI [18]. Zreaqat et al. observed that using twin blocks in Class II patients with retrognathic mandibles significantly increased upper airway volume without affecting nasopharyngeal dimensions [36]. Ghodke et al. also noted enhanced pharyngeal airway passage in 8- to 14-year-olds using functional devices like the Herbst appliance have shown improvements in both oropharyngeal and hypopharyngeal airway dimensions [37].

Activator appliances have also been investigated for their role in airway enhancement among paediatric patients. Medina et al. found that using activators in skeletal Class II cases improved airway dimensions and reduced disordered breathing events, ultimately supporting better sleep and quality of life [38].

Headgear, although used to address Class II malocclusion, tends to produce more dentoalveolar changes than skeletal modifications. While a few studies have raised concerns about its influence on the airway, most have found no significant negative impact [39]. Interestingly, upper molar distalization through headgear in non-growing patients has been associated with spontaneous widening of the mandibular arch. This may be due to altered tongue posture and positioning, which in turn influences the airway space, particularly as the base of the tongue forms the anterior wall of the oropharynx [18].

In addition to established approaches such as RPE and conventional MADs, recent orthodontic innovations have expanded the therapeutic scope for OSA. Miniscrew-assisted RPE (MARPE) has demonstrated measurable increases in nasal cavity volume and airway patency in skeletally mature patients, offering a non-surgical alternative to surgically assisted RPE (SARPE) [34-46]. Likewise, customized, titratable MADs designed with digital workflows allow for more precise mandibular positioning, improved patient comfort, and potentially better compliance [34,44]. Preliminary studies are exploring combined protocols, for example, MARPE with MADs, to achieve synergistic airway stability and symptom reduction, although robust long-term evidence is still needed [34]. Interdisciplinary collaboration between orthodontists and sleep physicians is recommended, in line with guidance from the American Sleep Association [17].

Mandibular Advancement (Invasive)

Surgical mandibular advancement is generally recommended for patients with severe OSA, particularly those who do not respond to CPAP therapy and present with notable maxillomandibular discrepancies. Orthognathic procedures can involve repositioning either the maxilla, the mandible, or both jaws simultaneously (bimaxillary surgery). When both jaws are surgically moved forward, the result is an expansion of the upper airway in both the lateral and anteroposterior dimensions, making this method highly effective in improving airway patency [18]. Typically, the surgical technique includes a Le Fort I osteotomy for the maxilla and bilateral sagittal split osteotomies for the mandible. By physically altering the facial skeletal framework where the pharyngeal muscles and tongue are anchored, this approach enhances the space available in the upper airway.

This type of surgical correction may be used on its own or in conjunction with orthodontic treatment. There are three common planning protocols: (i) surgery without orthodontic preparation, (ii) a surgery-first protocol followed by postoperative orthodontics, or (iii) traditional sequencing with pre-surgical orthodontics leading up to the operation. The third approach is most appropriate for patients who can tolerate CPAP during the preparatory orthodontic phase [11].

Despite the proven effectiveness of bimaxillary advancement in many cases, surgical outcomes can vary, and not all procedures lead to positive results. For instance, mandibular setback surgery to correct prognathism may actually reduce airway volume, predisposing some patients to OSA [40]. Henkin et al. described a case involving surgery first repositioning of the mandible in a patient with Class III malocclusion, which unexpectedly led to the onset of OSA symptoms; the issue was later resolved through additional procedures, including genioplasty and mentoplasty [40]. The surgical outcome was monitored through sleep studies and clinical evaluations to assess improvement [41].

Beyond skeletal modification, maintaining the tone and activity of pharyngeal muscles, particularly the genioglossus, is vital for airway stability. As an alternative to CPAP, hypoglossal nerve stimulation has emerged as a promising, albeit invasive, therapy for patients with moderate to severe OSA [1]. This treatment works by activating the hypoglossal nerve to protrude the tongue forward during inhalation, helping prevent airway collapse.

The system involves a pulse generator implanted in the chest (just below the collarbone), with a stimulation lead attached to the main branch of the hypoglossal nerve via a neck incision. Another sensor lead, responsible for detecting breathing effort, is inserted in the chest wall near the fourth intercostal space using a tunnelling method. Patients use a handheld remote to activate the system before sleep and deactivate it upon waking. Clinical data suggest significant reductions in both AHI and oxygen desaturation index with this therapy; however, its high cost and potential side effects, including tongue pain, discomfort from stimulation, and transient weakness, must be carefully considered [18].

SARPE and MARPE

An additional surgical option for addressing airway-related concerns is SARPE, typically recommended for individuals with pronounced palatal constriction. By widening the upper jaw, SARPE has been associated with improved sleep quality, specifically by lowering daytime fatigue, minimizing backward tongue displacement, and enhancing oxygen saturation during sleep [42]. However, the evidence is mixed. In a study by Pereira et al., the effects of SARPE were assessed in 15 patients with transverse maxillary deficiency. The results revealed no significant changes in upper airway volume or area following the procedure [43].

Another emerging approach is MARPE, which utilizes temporary anchorage devices (mini-screws) to apply orthopaedic forces directly to the palatal bones, thereby separating the fused mid-palatal suture [44]. Yi et al. reported that MARPE treatment led to an 8.48% increase in nasopharyngeal volume in adolescents aged 15-19 years with narrow maxillary arches [45]. Supporting these findings, Brunetto et al. evaluated 20 patients under the age of 18 who received MARPE therapy and compared them with 12 untreated controls [46]. The treated group demonstrated significant improvements in respiratory volume, a notable 65.3% reduction in AHI, and self-reported improvements in both sleep quality and reduction of daytime tiredness.

Although these preliminary findings are promising, further research is necessary to confirm the long-term benefits and broader applicability of SARPE and MARPE in managing OSA-related cases

Extraction and Non-Extraction

The extraction of first premolars is a routine orthodontic procedure conducted when the arch is narrow or anterior teeth retraction is required [19]. As mentioned in a few studies, after the first premolar extraction, when maximum anchorage is used for anterior retraction, a shortening of the dental arch may occur, which can lead to more posterior displacement of the tongue with a consequent decrease in the upper airway dimensions [18,19,47].

In contrast, several researchers have concluded that the extraction of the first premolar does not have any impact on the upper airway. In their systematic review and meta-analysis, Papageorgiou et al. investigated the effect of first premolar extraction on the airway and did not find any significant differences between extraction and non-extraction patients in terms of minimum cross-sectional area of the upper airway [48]. Larsen et al. reviewed electronic health records in Minnesota and found that the subjects without missing premolars and with four missing premolars were both diagnosed with OSA [49]. Thus, extracting the first premolars doesn’t negatively influence the upper airway and is not one of the causes of OSA. Pliska et al. conducted a study to evaluate volumetric treatment changes for the extraction and non-extraction groups, which concluded that treatment changes in the volume or minimal cross-sectional area for all airway regions examined were not significantly different between the extraction and non-extraction groups [50]. 

Novel Techniques and Prospects

In recent years, the use of smartphones and digital devices to monitor sleep patterns has attracted considerable attention from both researchers and healthcare professionals. These technologies are now being explored as alternatives to traditional sleep studies, offering more accessible ways to assess sleep-related concerns at home [50]. However, the reliability of such tools is often dependent on how well users understand and operate them, which raises concerns about the accuracy of the data collected.

The American Academy of Sleep Medicine (AASM) has emphasized that Consumer Sleep Technology (CST), including Sleep Assessment Technology Devices (SATDs), should not be used for diagnosis or treatment planning unless they are officially approved by the United States Food and Drug Administration (FDA) [51]. At this stage, these devices are considered complementary tools rather than replacements for clinical evaluation. One of their more promising roles is in enhancing communication between patients and healthcare providers, serving as conversation starters or tracking aids in ongoing care [18].

In 2018, AASM released safety guidelines for patients interested in using CST tools for personal health insights. Due to the current lack of robust clinical validation, these devices are not suitable for medical decision-making [51].

A wide range of consumer-friendly apps and devices have since entered the market, aiming to provide continuous, low-cost home monitoring of sleep disorders like OSA. These include wearables (worn on the wrist, fingers, head, or chest) as well as non-wearable sensors placed near the body to remotely detect physiological and behavioural signals during sleep. Despite their popularity, most of these tools still lack scientific backing, and further studies are needed to establish their clinical reliability. That said, they hold valuable potential in research and large-scale data collection, particularly for sleep-related studies [52].

## Conclusions

OSA is a multifactorial condition that involves anatomical as well as functional components of the craniofacial structures. The close relationship between airway anatomy and the craniofacial complex, such as a narrow maxilla or a retruded mandible, highlights the crucial role of orthodontists in the management of OSA. The main orthodontic interventions include rapid maxillary expansion, which showed a positive impact, especially in children with narrow maxilla and OSA. Mandibular advancement appliances, both functional and orthopaedic, are also feasible options for OSA by advancing the mandible, as patient compliance is greater. Lastly, orthognathic surgery to modify the jaw has also shown promising results in patients with skeletal disharmony and OSA. That said, careful patient selection as well as case severity are required to opt for the surgical approach. Furthermore, studies with larger sample sizes are needed to evaluate the impact of RPE in adults and MADs in younger patients. Future research should also explore how surgical interventions can be improved to maximise the efficacy of the procedure. Early interceptive devices should be further evaluated so that anatomical imbalances can be altered during growth with minimally invasive instruments.
